# Machine Learning Analysis of Individual Tumor Lesions in Four Metastatic Colorectal Cancer Clinical Studies: Linking Tumor Heterogeneity to Overall Survival

**DOI:** 10.1208/s12248-020-0434-7

**Published:** 2020-03-16

**Authors:** Diego Vera-Yunca, Pascal Girard, Zinnia P. Parra-Guillen, Alain Munafo, Iñaki F. Trocóniz, Nadia Terranova

**Affiliations:** 1 Pharmacometrics & Systems Pharmacology, Department of Pharmaceutical Technology and Chemistry, School of Pharmacy and Nutrition, University of Navarra, Pamplona, Spain; 2 Merck Institute for Pharmacometrics, Merck Serono S.A., Switzerland, a Subsidiary of Merck KGaA, Darmstadt, Germany; 3 IdiSNA, Navarra Institute for Health Research, Pamplona, Spain

**Keywords:** cetuximab, individual tumor lesion dynamics, machine-learning, metastatic colorectal cancer, survival analysis, tumor size modeling

## Abstract

Total tumor size (TS) metrics used in TS models in oncology do not consider tumor heterogeneity, which could help to better predict drug efficacy. We analyzed individual target lesions (iTLs) of patients with metastatic colorectal carcinoma (mCRC) to determine differences in TS dynamics by using the ClassIfication Clustering of Individual Lesions (CICIL) methodology. Results from subgroup analyses comparing genetic mutations and TS metrics were assessed and applied to survival analyses. Data from four mCRC clinical studies were analyzed (1781 patients, 6369 iTLs). CICIL was used to assess differences in lesion TS dynamics within a tissue (intra-class) or across different tissues (inter-class). First, lesions were automatically classified based on their location. Cross-correlation coefficients (CCs) determined if each pair of lesions followed similar or opposite dynamics. Finally, CCs were grouped by using the K-means clustering method. Heterogeneity in tumor dynamics was lower in the intra-class analysis than in the inter-class analysis for patients receiving cetuximab. More tumor heterogeneity was found in KRAS mutated patients compared to KRAS wild-type (KRASwt) patients and when using sum of longest diameters *versus* sum of products of diameters. Tumor heterogeneity quantified as the median patient’s CC was found to be a predictor of overall survival (OS) (HR = 1.44, 95% CI 1.08–1.92), especially in KRASwt patients. Intra- and inter-tumor tissue heterogeneities were assessed with CICIL. Derived metrics of heterogeneity were found to be a predictor of OS time. Considering differences between lesions’ TS dynamics could improve oncology models in favor of a better prediction of OS.

**Electronic supplementary material:**

The online version of this article (10.1208/s12248-020-0434-7) contains supplementary material, which is available to authorized users.

## INTRODUCTION

Model-Informed Drug Discovery and Development (MID3) (1) has demonstrated its usefulness to improve drug development in several cases, including the oncology area and the modeling of tumor size (TS) (2,3). TS is often expressed as the sum of longest diameters (SLD) of the individual tumor lesions (iTLs) measurable and defined as “target lesions” at baseline, as described by Response Evaluation Criteria in Solid Tumors (RECIST) (4). Each patient presents multiple iTLs, which can be primary or metastatic and located in several organs or tissues. This means that all tumor lesions, regardless of their location and status, are reduced to this single SLD value, also called the total TS, at each assessment visit within a patient. The iTLs are assessed throughout the clinical study; the SLD is derived at each time point and then categorized to quantify the tumor response to the treatment (4). Observed or model-derived TS metrics, such as the early tumor shrinkage (ETS, relative reduction of total TS at certain time points) or time to tumor growth, have been shown to be predictors of overall survival (OS) (5).

As an oversimplifying measure of cancer progression, the use of total TS may cause a loss of information with respect to tumor heterogeneity that could carry valuable information to predict disease progression, drug efficacy, and OS accurately. Indeed, differences in iTLs dynamics could reflect tumor heterogeneity: tumor lesions may present several clones which evolve in a Darwinian process at different rates, showing different phenotypes and drug resistances (6). Tumor heterogeneity is one of the factors involved in tumor resistance (7) and tumor metastasis (8). Recently, some modeling works in the oncology area successfully included tumor heterogeneity (9) and related this lesions’ variability to OS (10). Not including tumor heterogeneity into TS models may hide iTLs resistance development or other differences in response to treatment. This is not only due to the different clonal phenotypes but also due to the different tumor microenvironments in which the lesion has appeared.

In a previous work (11), the analysis of iTLs was performed to assess differences in lesions dynamics by using the Classification Clustering of Individual Lesions (CICIL) methodology, which integrates knowledge from signal processing and machine learning (ML). Results were used to then inform decisions about building a longitudinal model for iTLs or for total TS. The work presented in this paper continues and expands the innovative approach started by Terranova *et al.* by applying CICIL to four clinical studies in which cetuximab was administered in different combination therapies. TS data from these studies were analyzed either to assess the iTL dynamics between different organs or anatomic regions or to determine tumor dynamics differences within an organ or tissue. Furthermore, several comparisons between groups of patients based on differences in gene mutations and tumor metrics were performed. The impact of tumor heterogeneity on the clinical outcome was also assessed.

The objectives of this work were as follows: (i) to determine tumor heterogeneity in lesion dynamics using iTLs, (ii) to compare iTLs dynamics from patients based on genetic mutations (KRAS) and different TS metrics, and (iii) to apply these results in survival analyses of considered clinical trials.

This approach was applied to four metastatic colorectal cancer (mCRC) clinical studies. CRC is any kind of cancer which affects the colon or rectum. More than 1.8 million new cases and 881,000 deaths related to CRC were estimated to occur in 2018 (12). If only mCRC is considered, the main therapy for many years was 5-fluorouracil (5-FU) with folinic acid (FA). This therapy regimen showed a poor response rate (20%) and a median OS of about 6 months (13). Newer chemotherapy drugs, like irinotecan and oxaliplatin, improved the response rate to 31–34% and the median OS to approximately 24 months (13,14). Monoclonal antibodies have provided new weapons to fight mCRC. One of them is cetuximab, a monoclonal antibody that targets the epidermal growth factor receptor (EGFR). The EGFR is involved in survival, proliferation, tumor invasion, and tumor immune evasion. It has been observed that patients with *RAS* mutations, including mutations of the *KRAS* and *NRAS* genes, present poorer response to EGFR inhibitors (15) such as cetuximab, which is the drug studied in this work. According to intention-to-treat (ITT) populations in considered clinical trials, only information about KRAS status was available and was accounted in our assessments.

To help the reader, a list of abbreviations used throughout the text is reported in the Supplementary material.

## METHODS

### Trials and Data

TS data of iTLs in patients with EGFR expressing mCRC were obtained from four clinical studies: (i) CRYSTAL (Cetuximab combined with iRinotecan in first-line therapY for metaSTatic colorectAL cancer, electronic medical record 62202-013) (16), (ii) APEC (Asia Pacific non-randomized, open-label phase II study evaluating the safety and efficacy of folinic acid (FA) + 5-fluorouracil (5-FU) + irinotecan (FOLFIRI) plus cetuximab (Erbitux) or FA + 5-FU + oxaliplatin (FOLFOX) plus cetuximab as first-line therapy in subjects with KRAS wild-type (KRASwt) metastatic Colorectal cancer, electronic medical record 62202-505) (17), (iii) Study 045 (electronic medical record 62202-045) (18), and (iv) OPUS (OxaliPlatin and cetUximab in firSt-line treatment of mCRC, electronic medical record 62202-047) (19). Table I describes the main features of these four clinical studies. More detailed information about the clinical studies is presented in the Supplementary material. The ITT populations included RAS unselected subjects in CRYSTAL, Study 045, and OPUS studies and KRASwt subjects in the APEC study.

**Table I:** Overview of Considered Cetuximab mCRC Clinical Studies

Study	Study phase	No. of patients	Study arms	Cetuximab dosing schedule
CRYSTAL	III	1198	FOLFIRI (*N* = 599) *vs* FOLFIRI + cetuximab (N = 599)	Initial: 400 mg/m^2^, then 250 mg/m^2^ weekly
APEC	II	289	Investigators’ choice of FOLFIRI + cetuximab (*N* = 101) or FOLFOX + cetuximab (*N* = 188)	500 mg/m^2^, every 2 weeks
Study 045	I	62	Cetuximab weekly (*N* = 13) *vs* Cetuximab every 2 weeks^*a*^ (*N* = 49)	Initial: 400 mg/m^2^, then 250 mg/m^2^ weekly (A) or 400–700 mg/m^2^ biweekly (B)
OPUS	II	337	FOLFOX (*N* = 168) *vs* FOLFOX + cetuximab (*N* = 169)	Initial: 400 mg/m^2^, then 250 mg/m^2^ weekly

*FOLFIRI* folinic acid + 5-fluorouracil + irinotecan, *FOLFOX* folinic acid + 5-fluorouracil + oxaliplatin

^*a*^After week 6, all patients are administered FOLFIRI every 2 weeks + their cetuximab dosing regimen, weekly or every 2 weeks depending on the group the patients were in

### Tumor Size Quantification

Lesions TS was quantified either by computed tomography scan or magnetic resonance imaging. At baseline, iTLs were defined as measurable lesions representative of all involved organs, with a maximum of 5 lesions per organ and 10 lesions in total. According to studies protocols, the same method of assessment and the same imaging technique was used to characterize each identified and reported lesion at baseline and at each subsequent imaging time point. In the CRYSTAL, 045, and OPUS studies, iTLs were bidimensionally evaluated by using the modified WHO criteria (20,21), which quantifies the total TS by measuring the longest and perpendicular diameters of iTLs and then, deriving the so-called SOPD, sums of the products of diameters. In APEC, the assessment of response was performed according to RECIST (4,22) which uses the sum of longest diameters SLD of iTLs as a measure of total TS. Thus, unidimensional measurements were collected for iTLs in APEC, while bidimensional measurements were available for the other studies (23). In addition to the recorded TS measures over time, information about the lesion site was collected for all iTLs as text in the case report form for all studies. The lesion type was also coded as follows: primary, metastatic, or node. Calculated SLD or SOPD of lesions selected as target lesions and the recorded information on non-target lesions and new lesions were used to derive response and progression outcomes throughout the studies.

### Dataset Preprocessing

iTL data from the four clinical studies was extracted from the clinical database. Patients with only the tumor assessment at baseline as well as tumor data measured after tumor surgery procedures were excluded from the analysis. The main CICIL analyses presented in this work used the longest diameter as a single TS metric being available across all studies. In the three studies having bidimensional measures of TS available for each iTL, CICIL was re-run for comparisons of results with the two metrics.

### CICIL Methodology

The previously developed CICIL methodology, implemented in a Java-based platform, was used to evaluate the similarities or differences between iTL dynamics (inter-organs or intra-organ) (11). This methodology consists of 3 steps: (i) iTLs are classified based on their location and type described by expert physicians, (ii) cross-correlation (CC) values are estimated to assess the degree of similarity between dynamics of two lesions, and (iii) similar cross-correlation values are automatically grouped into clusters using the K-means clustering method (24). This approach can be easily applied to either the bi-dimensional product (WHO criteria), the longest diameter (RECIST criteria) or any future emergent volumetric measurement provided by progresses in tumor imaging and/or tumor size collection. Thus, we could analyze lesion sizes regardless of tumor evaluation criteria for diagnosis of progression of disease adopted in the studies.

**Fig. 1:**
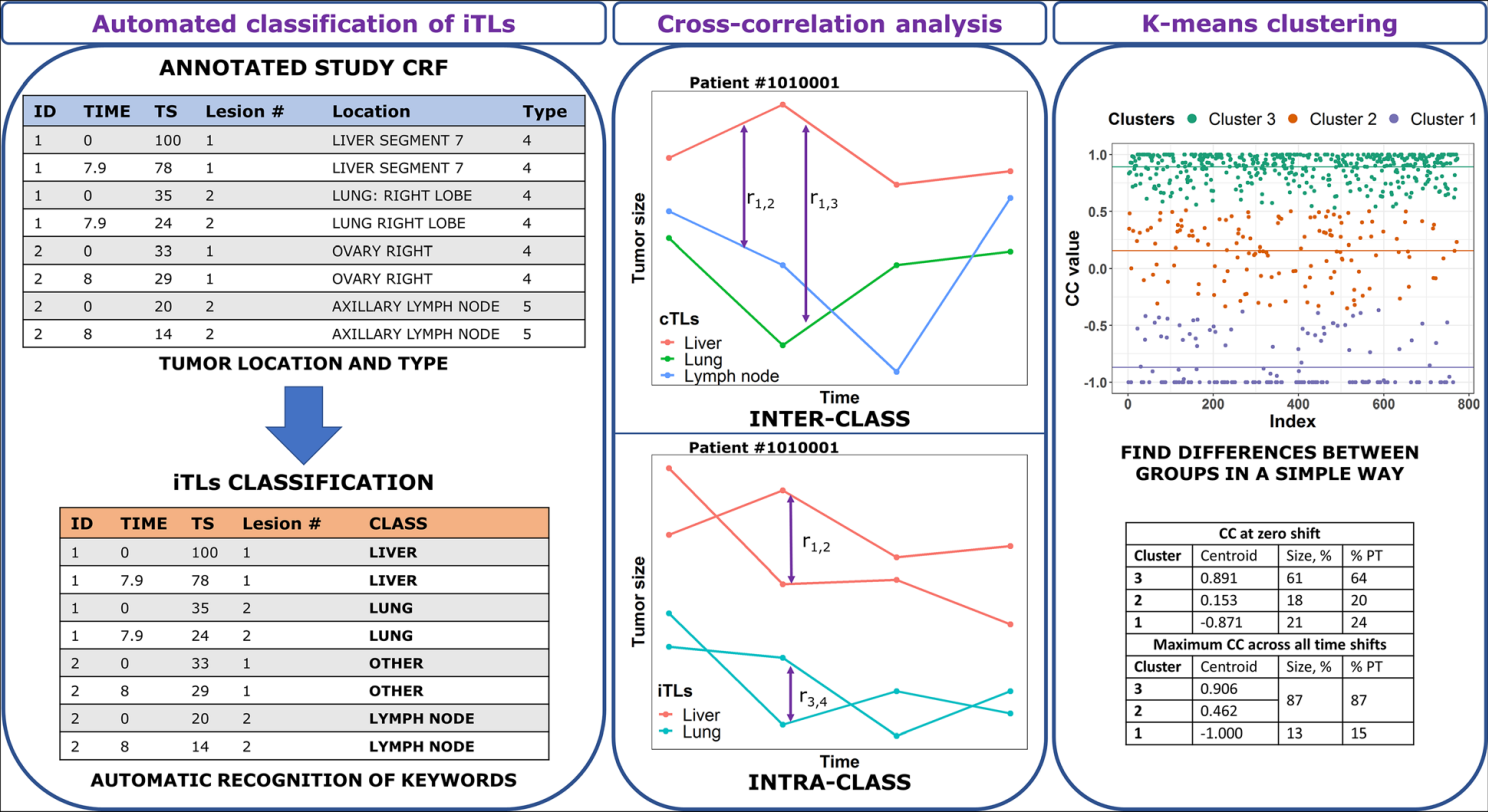
CICIL methodology overview. Clinical trials present in their case-report forms (CRFs) information about tumor size, time, tumor location, and tumor type of individual tumor lesions (iTLs). This data can be used to classify these iTLs. Then, the degree of similarity between the time-course of those lesions in a patient can be computed with the cross-correlation, both between lesions belonging to different classes (cTLs, inter-class) and between iTLs within a class (intra-class). Similar cross-correlation coefficients (CCs) are grouped by applying the k-means clustering technique to find differences between groups of CCs

Two kinds of CICIL analyses were performed: inter-class analysis and intra-class analysis. On one hand, the inter-class analysis uses the sum of lesions TS within each patient’s individual organs defined in the CICIL classification. They are called class-related target lesions (cTLs). Therefore, each patient shows a single cTL for each organ/tissue. Then, the CC value of the pairs of cTLs the patient presents with is computed to assess the difference in lesion dynamics between organs or tissues. This analysis then includes only patients presenting iTLs in more than one tissue. On the other hand, the intra-class analysis uses the iTLs from a single organ or tissue to compute the CC values for the pairs of iTLs in that organ or tissue. This is performed to determine the differences in lesion dynamics between iTLs within the same patient’s individual organ or tissue. Only patients presenting more than one iTL within the same organ are included in the analysis of that specific class. Figure 1 shows an overview of the CICIL methodology.

#### Step 1: Rule-Based Classification

iTLs were classified according to the same methods described in (11), which were defined for two other mCRC clinical studies*.* Several keywords were defined for each class in the classification text file of CICIL. These were based on the recorded tumor location and anatomical and physiological features observed on tumor lesions of these organs. The CICIL platform performed the automated classification process by recognizing the defined keywords and locations of each lesion in the extracted clinical dataset. Before running the classifier tool, missing or wrong organ information was checked (and corrected, if needed) during dataset preprocessing. Lesions with missing organ information were classified in the general “Unclassified lesions” class.

#### Step 2: Cross-correlation Analysis

In order to automatically compare hundreds of TS dynamics, while keeping information on the lesion dynamics across the whole study, the non-parametric comparison of two TS time courses, treated as time series, was performed by estimating the CC (25). The CC values can range from − 1 to 1. CC values equal or close to 1 indicate similar tumor dynamics for the two compared lesions. CC values equal or close to − 1 indicate opposite profiles. In contrast, CC values close to 0 indicate undefined relationships (i.e., not distinguishable) or small trends towards similarity or difference depending on the sign and absolute value of the CC. Thus, the CC value obtained from two TS dynamics was used as a metric of similarity or difference between such lesion dynamics.

CC values were calculated without accounting for any delay between TS dynamics (zero-time shift) as well as at shifting one of the lesions over the other (and vice versa) in time to maximize their CC value. The number of assessments per lesion allowed us to check up to 12 time shifts in both directions. Details on CC calculations are reported in (11).

#### Step 3: K-Means Clustering

K-means clustering is a ML unsupervised clustering technique (24). As the last step of CICIL, this method was used to group the different CC values into clusters. Then, differences between these groups or clusters could be assessed based on the centroid value (the average CC value of each cluster) and the percentage of lesion comparisons (i.e., CCs) and patients in each cluster. Two arbitrary cutoffs were established for easy interpretation of results: clusters with centroids below − 0.35 were considered as an indication of different lesion dynamics, whereas those with centroids above 0.35 were considered as pointing to similar dynamics. Clusters with values in between were considered as suggesting undefined relationships.

Beforehand, the number of clusters was selected with the elbow method which allows to assess and then choose the smaller number of clusters having a lower sum of squared errors (SSE) (26). If SSE *versus* the number of clusters were plotted, the arm would be the plotted line and the elbow would correspond to the optimal number of clusters.

### Survival Analysis

Overall survival time data were extracted from the four studies in order to perform survival analyses and to assess whether results from the CICIL analyses could be used as predictors of survival time. Kaplan-Meier plots were obtained, log-rank tests were computed, and a Cox proportional hazards model was fitted to the data. One of the tested predictors was the median CC for the inter-class analysis, which was computed for each cetuximab arm patient as the median of CC values at zero-time shifts obtained from the different lesion pairs (e.g., liver-lung or liver-node) within a patient. Given its relationship with patients’ response, the KRAS status was also tested as a predictor.

### Software

Dataset preprocessing was carried out in R version 3.5 (27) by importing the clinical SAS® dataset with the function *read_sas* from the package *haven* 2.2.0 (28). The CICIL methodology was performed in its Java-based software version 1.0.4. (11). For the survival analysis, R was used to run statistical tests and models, along with the *survival* package (29).

## RESULTS

**Fig. 2:**
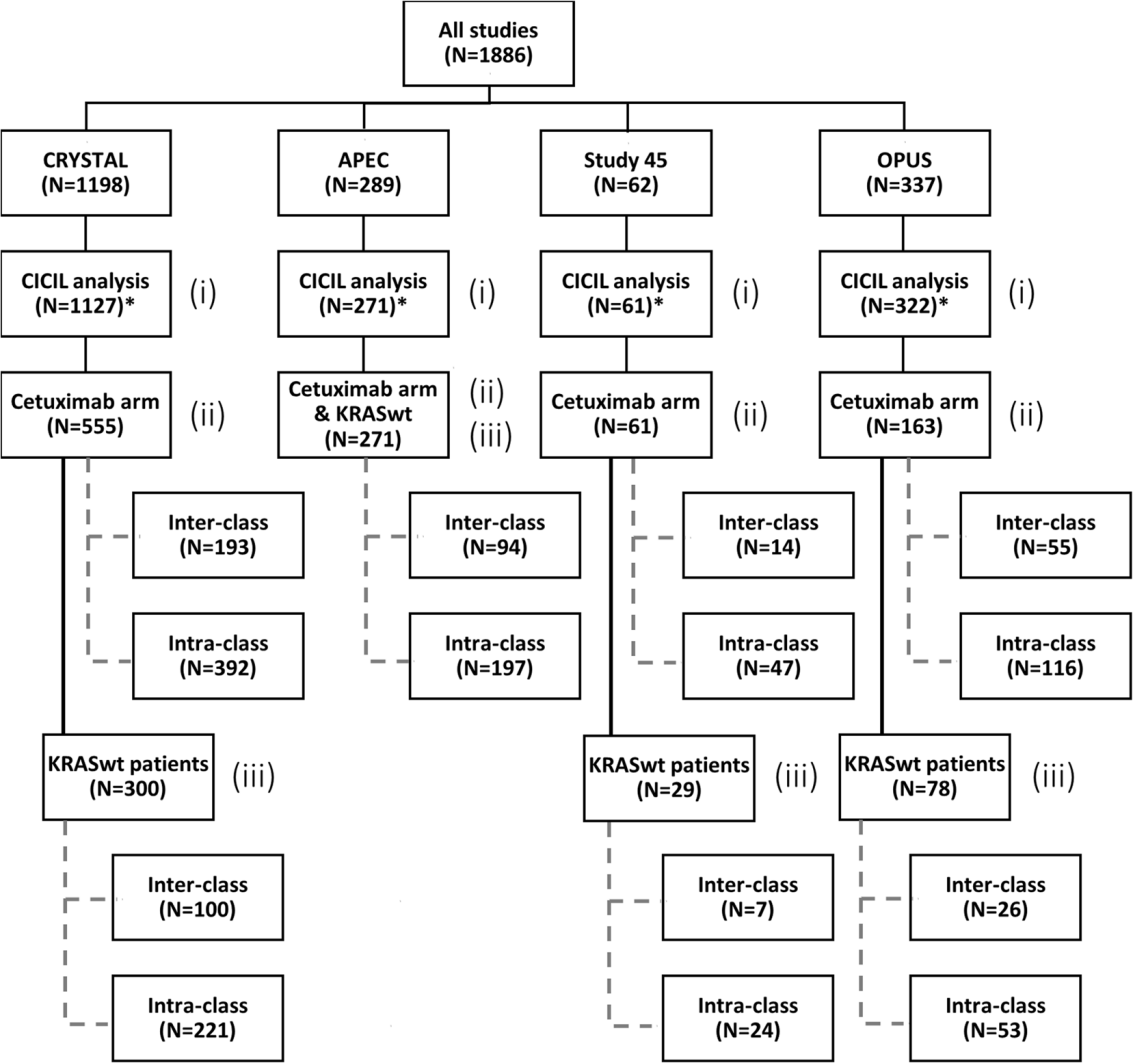
Tree diagram showing the distribution of patients across different subsets of CICIL analyses. Analyses on different subsets of patients were performed: (i) all patients, (ii) all patients receiving cetuximab either as monotherapy or in combination (cetuximab arm), and (iii) KRASwt patients receiving cetuximab. All APEC patients were KRASwt. *Not all patients were able to enter the CICIL analysis. Those without more than one tumor size (TS) assessment or with missing organ information were excluded

For the CICIL analysis, 1781 patients from the four previously described clinical studies were included: 1127 patients from CRYSTAL, 271 patients from APEC, 61 patients from Study 45 and 322 patients from OPUS. Separate CICIL analyses were performed for each study by considering different subsets of data: (i) all patients, (ii) patients receiving cetuximab, and (iii) KRASwt patients receiving cetuximab. All patients from the APEC and Study 045 studies received cetuximab. Figure 2 shows a tree diagram with the number of patients considered at each stage of this work.

### Classification

iTLs were classified into eight classes based on the organ keywords and location information. This classification was adapted from the previous work by Terranova *et al.* (11) and found to be suitable for our clinical studies. The defined and adapted classes were as follows: “Liver”, “Lung”, “Lymph node”, “Other respiratory organs”, “Other digestive organs”, “Other specified organs”, “Primary lesions”, and “Unclassified lesions”. The “Primary lesions” class was populated with non-metastatic lesions, whereas “Unclassified lesions” class contained lesions without proper organ information.

Similarly to the previously described CICIL work (11), a second classification step was performed, as the “Other respiratory organs”, “Other digestive organs”, “Other specified organs”, “Primary lesions” and “Unclassified lesions” classes presented less than 30 patients. These classes with low numbers of patients were combined into the “Other” class. Table II shows the total number of cTLs (equal to the number of subjects with at least one lesion in that class) and the number of iTLs for each subset of data (all patients, patients who received cetuximab, and KRASwt patients who received cetuximab) across classes. The liver class represented 68% of all the iTLs measured across all studies. Lung and lymph node classes accounted for 12% for iTLs each one. The class Other only included 8% of iTLs.

**Table II:** Classification of Individual Target Lesions

Class name	CRYSTAL			APEC	Study 45		OPUS			All studies		
	ALL	Cetuximab arm	KRASwt	ALL	ALL	KRASwt	ALL	Cetuximab arm	KRASwt	ALL	Cetuximab arm	KRASwt
Liver	884 (2867)	434 (1420)	227 (745)	176 (525)	51 (153)	26 (89)	265 (774)	139 (421)	63 (197)	1376 (4319)	800 (2519)	487 (1546)
Lung	252 (521)	132 (262)	63 (113)	68 (155)	10 (22)	5 (7)	49 (85)	24 (43)	8 (12)	379 (783)	234 (482)	141 (284)
Lymph node	261 (488)	110 (193)	62 (110)	72 (141)	4 (7)	0	60 (113)	33 (63)	19 (37)	397 (749)	219 (404)	148 (278)
Other	250 (319)	121 (151)	73 (89)	74 (102)	12 (12)	6 (6)	70 (85)	36 (42)	21 (25)	406 (518)	243 (307)	170 (218)
Total	1647 (4195)	797 (2026)	425 (1057)	390 (923)	77 (194)	37 (102)	444 (1057)	232 (569)	111 (271)	2540 (6369)	1496 (3712)	946 (2326)

The number of class-related target lesions (cTLs, sum of lesions of a class for a patient) is presented in the table. Individual target lesions (iTLs, 1 or more than 1 per patient) are shown in parenthesis. ALL, target lesions from all patients included in the CICIL analysis. Cetuximab arm, patients who received cetuximab either as a monotherapy or in combination with chemotherapy: FOLFIRI (folinic acid + 5-fluorouracil + irinotecan) or FOLFOX (folinic acid + 5-fluorouracil + oxaliplatin). KRASwt, target lesions from KRASwt patients only. All patients in APEC and Study 45 studies received cetuximab; thus, the “Cetuximab arm” columns are omitted. APEC only included KRASwt patients: in this case only the “ALL” column is shown

### Inter-class Analysis

#### All Patients

Only patients with more than one class could be included in the inter-class analysis, so this reduced the number of available cTLs for the analysis to 926 cTLs (from 404 patients) in the CRYSTAL study, 215 cTLs (from 94 patients) in the APEC study, 30 cTLs (from 14 patients) in Study 045, and 220 cTLs (from 99 patients) in the OPUS study.

When data from the four studies were analyzed together, three clusters were used. About 61% of lesion pairs (from 64% of patients), grouped into cluster 3, showed similar lesion dynamics. Cluster 2 contained 18% of comparisons. This cluster presented a small positive correlation between lesions. Remaining comparisons were included into cluster 1, with a centroid close to − 1 indicating that those lesions presented different dynamics. When time shifts were taken into account, the percentages of lesion comparisons and patients within clusters with similar dynamics or positive correlation (clusters 2 and 3) increased to 87%. Thus, less tumor heterogeneity across classes was suggested when taking into account time shifts of lesions dynamics.

#### Comparison: Cetuximab Arm Patients *Versus* Non-cetuximab Arm Patients

**Fig. 3:**
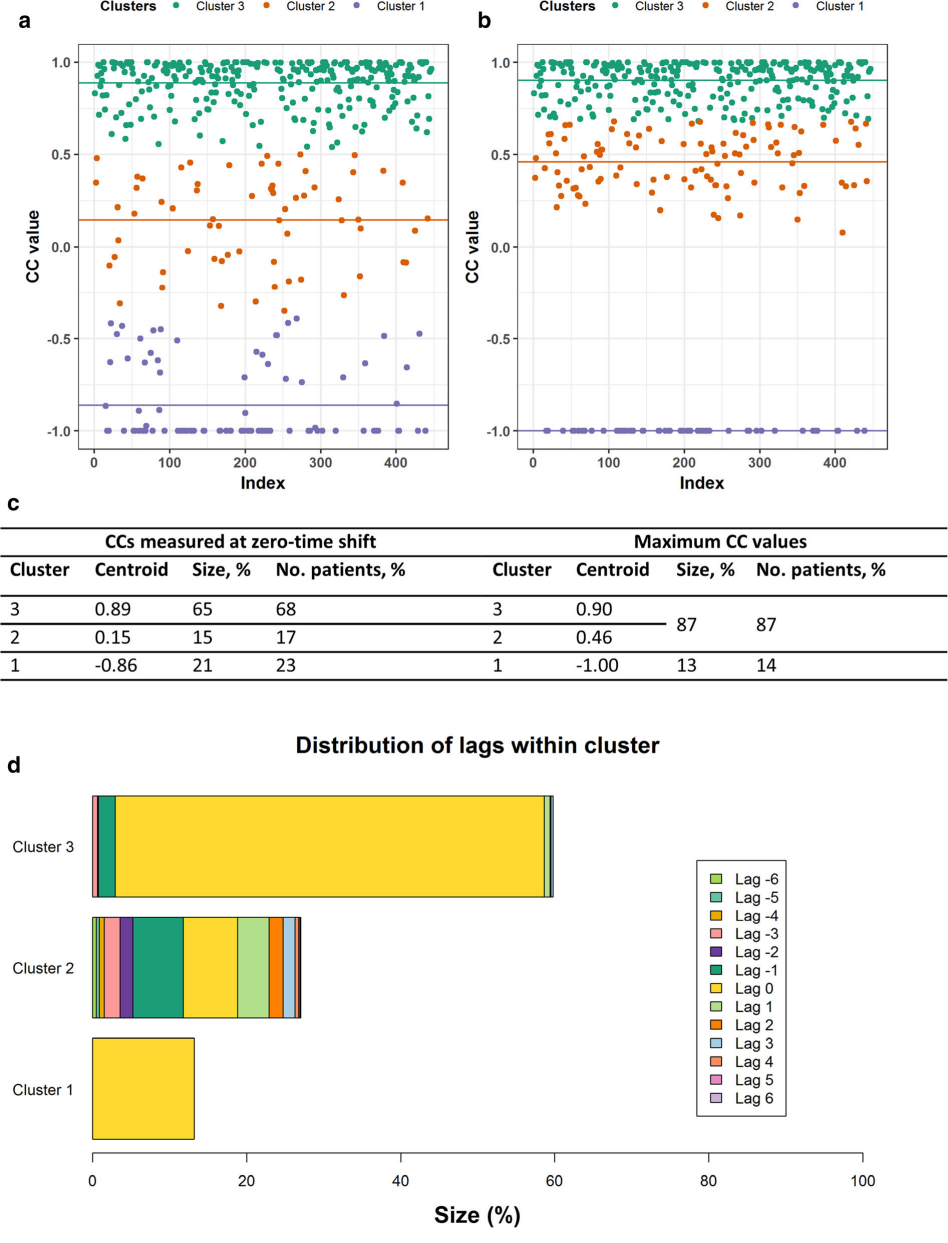
Inter-class analysis results for the combined data from the four clinical studies for those patients who received cetuximab, either as a monotherapy or in combination with the FOLFIRI (folinic acid + 5-fluorouracil + irinotecan) or FOLFOX (folinic acid + 5-fluorouracil + oxaliplatin) regimens. Results at zero-time shift (**a**) and when maximum cross-correlation coefficients (CC) values are achieved (**b**) are shown. Horizontal lines show the centroids for each cluster. Table in **c** details the results of this inter-class analysis for patients who were administered cetuximab. If the cluster centroid value was above 0.35, CCs from that cluster were considered to show similar dynamics and summed to CCs from cluster 3. Note that patient percentages accounted for more than 100%, because a single patient can have lesion pairs in different clusters. **d** The distribution of time shifts or lags at which the maximum CCs were obtained, within each cluster

Patients in the cetuximab arm were used to look for potential differences from patients not receiving cetuximab. The optimal number of clusters was 3. Larger percentages of lesion pairs (65%) and patients (68%) in the cetuximab arm were found in cluster 3 compared to the group of patients not receiving cetuximab (56% lesion pairs from 58% of patients). Nevertheless, the percentage of lesion pairs (21%) and patients (23%) in cluster 1 for the cetuximab group was similar to those in the group not receiving this therapy (21% lesion pairs from 25% of patients, respectively). This points to a similar tumor heterogeneity in lesion dynamics between the two subgroups. Results from the CICIL analysis on patients receiving cetuximab are shown in Fig. 3. Inspection of distributions of time shifts at which the maximum CC values were achieved within cluster 3 suggests good synchronicity (59% of maximum CCs at zero-time shift) between similar cTLs dynamics. Cluster 2 showed that small shifts (from − 1 to + 1) accounted for most of the maximum CC values in this cluster. Finally, cluster 1 presented all maximum CC values at zero-time shift. Indeed, these came from lesion pairs with only two tumor assessments, thus not allowing any series shift in time. Overall, 23% of patients in the cetuximab arm presented different lesion dynamics at zero-time shift, but if time shifts are considered (especially small lags like ±1, as stated above), only 14% of patients showed opposite lesion dynamics. An illustrative representation of the impact of small time shifts on CC is provided for cTLs with different CC values at zero-time shifts in Supplementary Figure S1.

#### Comparison: KRAS Wild-Type Patients *Versus* KRAS Mutated Patients

The CICIL analysis was also performed on KRAS subsets of patients to compare KRASwt *versus* KRAS mutated (KRASmut) lesion dynamics. Results based on the combined dataset of the four clinical studies showed that more similar lesion dynamics were found in patients with KRASwt mCRC lesions (72% lesion pairs belonging to 74% of patients) than in patients with KRASmut lesions (52% lesion pairs belonging to 57% of patients). Cluster 1 (different lesion dynamics) included 16% lesion pairs from 18% of patients in the KRASwt group and 29% of lesion pairs from 31% of patients in KRAS mutated group, respectively.

The same pattern was observed when the analysis was performed on the CRYSTAL and OPUS studies separately. No conclusions can be drawn for APEC and Study 045 studies: APEC had no KRASmut patients and Study 045 had a low number of eligible patients (14). See supplementary Table SI for more details.

#### Comparison: TS Measured as Product of Diameters *Versus* TS Measured as Longest Diameter

For the three studies with bidimensional TS measurements available, CICIL was re-run by using the SOPD of cTLs. Results were then compared to those previously obtained with SLD.

When all studies with SOPD measurements were analyzed together, similar percentages of lesion pairs (65% and 63% with SLD and SOPD, respectively) and patients (67% in both groups) were found in cluster 3. Percentages of lesion pairs (21%) and patients (23%) in cluster 1 were the same for both analyses. Nevertheless, the CRYSTAL study, the largest study in this work, presented larger percentages in cluster 3 with SOPD than with SLD measurements, both with respect to lesion pairs (60% *vs* 56%, respectively) and to patients (66% *vs* 62%, respectively). Cluster 1 included 22% *versus* 25% of lesion pairs and 23% *versus* 26% of patients, with SOPD and SLD, respectively.

Study 045 results displayed the same trend as CRYSTAL. However, due to the low number of lesion pairs, this result might not be as informative as the ones obtained in other studies. For the OPUS study, the percentages of lesion pairs and patients in clusters 2 and 3 were higher for SOPD than for SLD, but the same percentages were found in cluster 1 for both metrics. Overall, no major differences between SLD and SOPD results were shown for this study. See Supplementary Table SII for more details.

### Intra-Class Analysis

The CICIL methodology was also applied to assess the differences across iTL dynamics grouped into the same class. Therefore, CCs were computed across iTLs from the same class within the same patient. Supplementary Table SIII shows the intra-class analysis results for each class for the combined dataset of patients treated with cetuximab.

Overall, cluster 3 or both clusters 3 and 2 (provided the latter one presented a high-value centroid, i.e., greater than 0.35), included 71–88% of CC values for all the considered classes (“Liver”, “Lung”, “Lymph node”, and “Other”). This means most lesion pairs showed similar lesion dynamics within every class. When time shifts were considered, the three main classes (“Liver”, “Lung”, and “Lymph node”) decreased their percentage of CCs in cluster 1 (different lesion dynamics) from 8–23% at zero-time shift to 4–14% at maximum CC values. The “Other” class also decreased its percentage of CCs in cluster 1 from 29% at zero-time shift to 14% at maximum CCs.

### Application of CICIL Results into Survival Analysis

Following the inter- and intra-class analyses, we assessed whether a metric related to the CC values (representative of tumor heterogeneity) could be predictive of OS. The inter-class median CC for each patient was derived as a unique metric to then assign the patients to two groups: patients with a median CC value equal to or below 0.35 and patients with median CC value above 0.35. The CC value of 0.35 was selected because it was considered an appropriate conservative threshold. Setting a higher threshold would have meant that more CCs would have been considered to present different dynamics. Nevertheless, similar results were obtained when considering a threshold of 0.5.

**Fig. 4:**
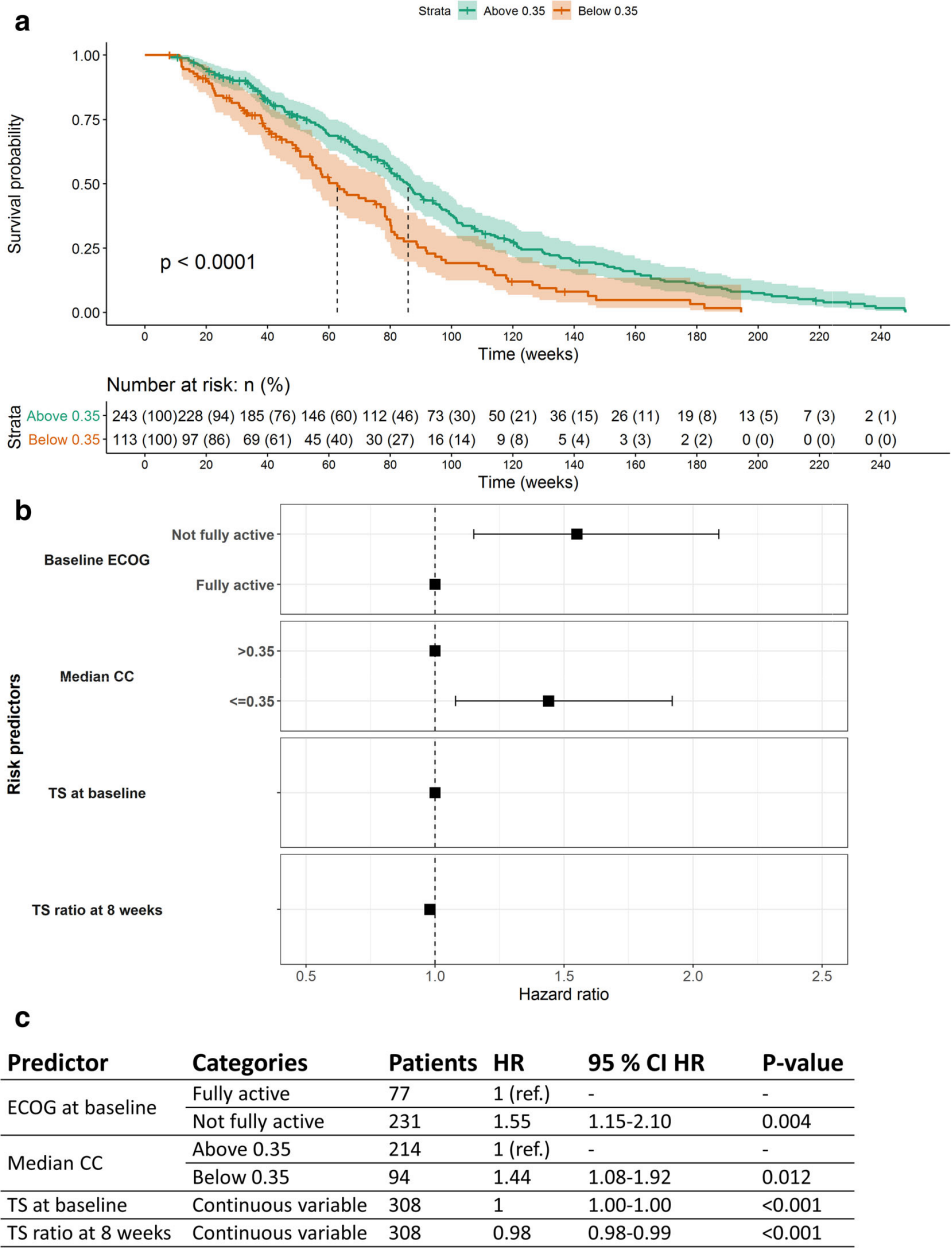
Survival analysis for the cetuximab arm of the meta-analysis pooled dataset. The Kaplan-Meier plot stratified by the median cross-correlation coefficient (CC) value is shown in (**a**). The median CC used as a predictor was categorized into two groups: median CC equal to or above (green) and below (orange) 0.35. The dashed lines show the median survival time for each group. The *p* value from the log-rank test is shown in the plot. Below the Kaplan-Meier plot, the number of patients (with percentages in parenthesis) at risk of death at each time is shown in a tabular format. The survival forest plot obtained from the final multivariate Cox proportional hazards model is shown in (**b**). ECOG score at baseline, tumor size (TS) at baseline, TS ratio at 8 weeks, and median CC predictors were assessed as predictors. Results are shown in **c** reporting the number of patients, hazard ratios (HR) along with their 95% confidence interval (95% CI), and *p* values (statistical significance) for each predictor included in the multivariate Cox proportional hazards model

A log-rank test was first performed on the meta-analysis pooled dataset, and the median CC value was found statistically significant (*p* value = 3.42 × 10^−5^). When each study was assessed individually and for any subset of data, only CRYSTAL showed statistically significant results (*p* value equal to 7.15 × 10^−5^). A Kaplan-Meier plot for the meta-analysis pooled data was then used to estimate median survival time in each group, which was 85.9 weeks (95% confidence interval (CI) 79.7–94 weeks) for those patients whose CC was above 0.35 and 62.7 weeks (95% CI 54.6–78.3 weeks) for the other group of patients. Figure 4a shows the Kaplan-Meier plot for the meta-analysis, stratified by the two CC groups. The potential confounding between KRAS status and heterogeneity quantified as median CC was also evaluated by deriving Kaplan-Meier plots stratified by the median CC (categorized as before) for KRASwt and KRASmut patients separately. In both groups, the median CC was found to be significant (*p* values equal to 0.0024 and 0.017 for KRASwt and KRASmut groups, respectively).

A multivariate Cox proportional hazards model was fitted to the meta-analysis pooled dataset using several risk predictor variables: KRAS status (categorized as KRASwt or KRASmut), ECOG performance score at baseline (as fully or not fully active), TS at baseline (continuous variable), and the ETS defined as the TS ratio at 8 weeks as a continuous variable (${\mathrm{TS}}_{\mathrm{ratio}}=\frac{\mathrm{TS}0-\mathrm{TS}8}{\mathrm{TS}0}$, where TS0 is the TS at baseline and TS8 is the TS at 8 weeks). These predictors had been already selected in a previous internal analysis based on total TS, and they were found to be statistically significant. The patient median CC value was added on top of this multivariate Cox model, both as a continuous and as a binary variable, to test its statistical significance as a predictor of risk. Results for the model containing the continuous median CC variable showed statistical significance for all predictors, except for the KRAS status which was significant in the univariate analysis but dropped from the final models (*p* value > 0.05). The *p* value of the median CC was 0.006 with hazard ratio (HR) of 0.74 (95% CI 0.60–0.92). This indicates that a CC value increase of one unit (e.g., from − 0.5 to 0.5, thus reducing tumor heterogeneity) would lead to a decrease in the risk of death of 26%. Although the KRAS status itself was not significant, the addition of an interaction term between the KRAS status and the continuous median CC resulted to be significant (*p* value = 0.022) and suggested that the effect of our heterogeneity measure was less pronounced in KRASmut patients with an average increase of the risk in this patient population by 60%. If median CC was introduced into the multivariate Cox model as a categorized binary variable reflecting the two groups (median CC above or equal to/below 0.35), the same predictors were significant. The median CC presented a HR of 1.44 (95% CI 1.08–1.92, *p* value = 0.012) for the group equal to or below 0.35 (i.e., more tumor heterogeneity). These results are shown in Fig. 4b and c. This means that patients with the median CC equal to or below 0.35 presented an average 44% increase in the risk of death. Of note, the lack of significance of KRAS status in the multivariate analysis was true only on this reduced CICIL analysis set which included only subjects having the median CC available (i.e., iTLs in more than one class). Indeed, *p* values smaller than 0.001 were obtained when running the analysis on all patients (including those without the median CC).

## DISCUSSION

Despite enriching data analysis in oncology, lesion heterogeneity has been often ignored or disregarded in the TS modeling, as also highlighted by the limited number of published works in this field (9,10). This is due to the complex models and methods needed to consider differences between organs or tissues, as well as the intra-tumor heterogeneity within an anatomic area. Models with such features are complex and computationally expensive. Thus, being able to efficiently assess and quantify the heterogeneity in iTLs TS dynamics prior to any modeling analysis is extremely valuable to inform and to guide the best modeling strategies. The recently proposed CICIL methodology addressed this objective by exploiting ML techniques to inform subsequent modeling steps (11). This approach provides a user-friendly and automated framework to quantify heterogeneity in tumor dynamics at different levels: (i) between iTLs within an organ and (ii) between lesions located in different tissues. Results can be used to make data-driven modeling decisions, for example, about the use of total TS *versus* iTLs TS, and about the level (within or across tissues) of tumor heterogeneity to be accounted for in the model.

In this work, we used CICIL to analyze TS data of patients’ iTLs from four mCRC studies investigating cetuximab treatment. The classification of iTLs considered the three main lesion classes “Liver”, “Lung”, “Lymph node”, and the general class “Other”. The low number of iTLs for the least frequent classes (i.e., “Other respiratory organs”, “Other digestive organs”, “Other specified organs”, “Primary lesions” and “Unclassified lesions”) did not allow us to assess intra-tumor heterogeneity in those classes separately and they had to be pooled into a single class called “Other”. Results indicated that the majority of iTLs were located in the liver (68%). Liver was also the most representative class in a previous analysis of two other cetuximab mCRC clinical studies (11). This is in line with available literature highlighting the liver as the most common site of metastasis in mCRC patients due to its anatomical position with respect to the portal circulation (30,31).

CICIL results from the intra-class analysis indicated little intra-tumor heterogeneity in TS dynamics of patients’ iTLs from the same classified tissue (less than 23% of iTL pairs at zero-time shift across the three main classes). Such results were obtained with a rich dataset including 863 patients and 2990 iTLs across studies which supports the robustness of the conclusions. Thus, we considered it conceivable to neglect intra-tumor heterogeneity in subsequent subgroup analyses performed in this work.

In the inter-class analysis, tumor heterogeneity between lesions located in different tissues (cTLs) was found to be higher. In particular, in the analysis based on the cetuximab arm, 36% of CCs or cTLs pairs (from 38% of patients) were found to follow different TS dynamics at zero-time shifts. This percentage is also marginally larger than the one (35% lesion pairs from 30 to 35% patients) obtained in a previous work (11), and it is mainly driven by the CRYSTAL study (44% lesion pairs from 45% patients) as the largest study in this work. When time shifts were considered, such percentage of CCs dropped to 13%. This points to the possibility that time delays could account for some of the observed tumor heterogeneity in the patient, e.g., a delayed response to the drug in some tumor tissues. It should be noted that in both analyses some patients presented iTLs with only two TS assessments, which made their CCs not as informative as those coming from lesions with more than two TS measurements.

Cetuximab patients with cTLs expressing KRASwt genetics showed less tumor heterogeneity in TS dynamics than KRASmut patients. Cetuximab, as an EGFR inhibitor monoclonal antibody, presents lower efficacy if lesions present with KRAS mutations (32). Therefore, we may relate this increased tumor heterogeneity in KRASmut patients to their reduced response to Cetuximab.

For those studies that measured the longest diameter and the perpendicular one, inter-class results obtained with the longest diameter were compared to those using the product of diameters as TS metric. The combined analysis from the pooled dataset did not suggest any differences between results obtained with SOPD and SLD metrics. Nevertheless, results for the CRYSTAL study showed smaller differences in cTL dynamics and then in tumor heterogeneity when SOPD was used as the TS metric (38% with SOPD *vs* 45% with SLD). This may point to a better characterization of tumor heterogeneity when bidimensional data are collected for this case study. Indeed, literature shows that such differences between TS metrics depend on the kind of cancer: in some cases, there are no differences between unidimensional or bidimensional measurements (33), while in other cases, there are significant differences between results obtained with these two metrics (34,35).

Tumor heterogeneity results obtained in the inter-class analysis were found to be a predictor variable of OS time in the form of median CC. Its significance was proven both alone (log-rank test and the Kaplan-Meier plot) and in combination with other known risk predictors (multivariate Cox proportional hazards model). Increased risk of death with increased heterogeneity was suggested by all tested models. Interestingly, the model including a significant interaction term between the heterogeneity metric and the KRAS status suggested a relevant decrease of risk (about 40%) with decreased heterogeneity in KRASwt patients, but a small decrease (about 3%) in KRASmut patients. This points to a reduced impact of heterogeneity in TS dynamics on risk for KRASmut patients which is however already associated with a lower treatment effect in this subpopulation. Pharmacokinetic-tumor size-overall survival relationships can be affected by immortal time and selection bias (36), in particular, in situations without dose-ranging data. As the estimation of our tumor heterogeneity measure is related to the number of tumor assessments the patient had over the study and this depends on the immortal time, the potential for immortal bias cannot be excluded. More sophisticated models including time-dependent covariates allowing a change in status over time may be tested in future works to overcome the potential for immortal time bias.

As one of the major outcomes of this work, such results further highlight the impact of tumor heterogeneity on tumor response and the importance of including it into survival analyses. Besides, modeling in the oncology arena could benefit from including measures of tumor heterogeneity data such as CC values. For example, the use of “tissue-agnostic” datasets in which individual lesions are considered and grouped based on the degree of similarity in their TS dynamics could improve the performance of TS models in favor of a better prediction of OS time.

## CONCLUSIONS

The CICIL outcome obtained from a large dataset of tumor measures was assessed with respect to different factors (genetic mutations, tumor metrics), and its direct link with a clinical endpoint was quantified. Comparisons between KRASwt and KRASmut patients indicated less heterogeneity in tumor lesions dynamics in the KRASwt subgroup which is known to well respond to Cetuximab treatment. The method used to measure the lesion TS did not lead to different results except for the single-study analysis of CRYSTAL where an apparent heterogeneity was compensated and reduced when including the perpendicular diameter and obtaining the SOPD. An increased risk of death with increased heterogeneity was suggested by all tested models, especially in KRASwt patients. A reduced impact of heterogeneity in TS dynamics on risk for KRASmut patients was indicated.

The identification of a new metric of tumor heterogeneity related to a clinical outcome as OS is a relevant finding in the exploration of TS metrics that can inform clinical development decisions. This further supports the use of continuous TS response metrics as endpoints in early clinical oncology studies in order to improve design efficiency.

## Electronic Supplementary Material

ESM 1 (DOCX 673 kb)

ESM 2 (PNG 301 kb)

High Resolution (TIF 657 kb)
