# Supplementary material for: Machine Learning Analysis of Individual Tumor Lesions in Four Metastatic Colorectal Cancer Clinical Studies: Linking Tumor Heterogeneity to Overall Survival
Source: AAPS J. 2020 Mar 16;22(3):58. doi: 10.1208/s12248-020-0434-7 (PMC7078147; doi:10.1208/s12248-020-0434-7)
Supplement: Supplementary file 1 — (DOCX 673 kb) [file 12248_2020_434_MOESM1_ESM.docx]

Supplementary material

**List of abbreviations**

| 5-FU | 5-Fluorouracil |
| --- | --- |
| APEC | Asia Pacific non-randomized, open-label phase II study evaluating the safety and efficacy of folinic acid (FA) + 5-fluorouracil (5-FU) + irinotecan (FOLFIRI) plus cetuximab (Erbitux) or FA + 5-FU + oxaliplatin (FOLFOX) plus cetuximab as first-line therapy in subjects with KRAS wild-type (KRASwt) metastatic colorectal cancer |
| CC | Cross-correlation coefficient |
| CICIL | ClassIfication clustering of individual lesions |
| CRYSTAL | Cetuximab combined with irinotecan in first-line therapy for metastatic colorectal cancer |
| CT | Computed tomography |
| cTL | Class-related target lesions |
| DLT | Dose-limiting toxicity |
| EGFR | Epithelial growth factor receptor |
| ETS | Early tumor shrinkage |
| FA | Folinic acid |
| FOLFIRI | Folinic acid + 5-Fluorouracil + irinotecan |
| FOLFOX | Folinic acid + 5-Fluorouracil + oxaliplatin |
| HR | Hazard ratio |
| iTL | Individual tumor lesion |
| ITT | Intention-to-treat |
| KRASmut | KRAS mutated |
| KRASwt | KRAS wild-type |
| mCRC | Metastatic colorectal cancer |
| MID3 | Model-informed drug discovery and development |
| ML | Machine learning |
| MRI | Magnetic resonance imaging |
| MTD | Maximum tolerated dose |
| OPUS | Oxaliplatin and cetuximab in first-line treatment of mCRC |
| OS | Overall survival |
| RECIST | Response evaluation criteria in solid tumors |
| SLD | Sum of longest diameters |
| SOPD | Sum of the products of diameters |
| SSE | Sum of squared errors |
| TS | Tumor size |

**Clinical studies: detailed description**

CRYSTAL was a phase III study with the objective to compare the FA + 5-FU + irinotecan (FOLFIRI) regimen versus the FOLFIRI regimen plus cetuximab. 1198 patients were enrolled in this study. Cetuximab’s initial dose was set to 400 mg/m^2^, and the following doses were of 250 mg/m^2^ weekly. After the administration of cetuximab, irinotecan was administered at a dose of 180 mg/m^2^ every 14 days, followed by FA (400 mg/m^2^ racemic formulation or 200 mg/m^2^ for the L-form formulation) and 5-FU (bolus of 400 mg/m^2^, then a continuous infusion of 2400 mg/m^2^ over 46 hours). iTLs were measured at study baseline, every 8 weeks and at the end of the study.

APEC was a phase II study which included 289 patients from the Asia-Pacific region. Patients were administered either FOLFIRI plus cetuximab or FA + 5-FU + oxaliplatin (FOLFOX) plus cetuximab to assess their efficacy and safety. The dose of 500 mg/m^2^ was selected for cetuximab, and it was administered every two weeks. Oxaliplatin was administered every 14 days using a dose of 100 mg/m^2^. Irinotecan, FA and 5-FU doses and dosing schedules were the same as for the CRYSTAL study. iTLs were measured at study baseline, every 8 weeks and at the end of the study.

Study 045 was a phase I study with the objective to quantify the maximum tolerated dose (MTD) for cetuximab. 62 patients were recruited for this study. FOLFIRI plus cetuximab regimen was administered. FOLFIRI doses were the same as in the previous studies, and they were administered every two weeks. FOLFIRI was not administered during the first 6 weeks to test Cetuximab as a monotherapy. At week 7, the dosing regimen consisted of Cetuximab + FOLFIRI. Cetuximab was administered in a different schedule depending on the group the patient was assigned to: weekly for the control group or every two weeks for the dose escalation groups. For the control group, an initial dose of 400 mg/m^2^ was followed by weekly doses of 250 mg/m^2^. The four experimental groups received escalating cetuximab doses of 400 mg/m^2^ every 2 weeks, 500 mg/m^2^ every 2 weeks, 600 mg/m^2^ every 2 weeks and 700 mg/m^2^ every 2 weeks. Individual patients were sequentially assigned to one of these five groups considering whether dose-limiting toxicity (DLT) had been observed at the previous dose level. iTLs were measured at study baseline, every 6 weeks and at the end of the study.

OPUS was a phase II study designed to evaluate whether the efficacy of FOLFOX plus cetuximab was superior to the efficacy of FOLFOX alone. 337 patients were enrolled in this study. Cetuximab, FA and 5-FU dosing schedules were the same as the one from the CRYSTAL study. The oxaliplatin dose was set to 85 mg/m^2^ every 2 weeks. Target tumor lesions were measured at study baseline, every 8 weeks and at the end of the study.

Supplementary tables

**Inter-class analysis results: KRASwt versus KRASmut patients for each study**

Table SI. Inter-class analysis results from KRASwt and KRASmut patients are reported side-by-side for each study.

| **CRYSTAL** | | | | | | | |
| --- | --- | --- | --- | --- | --- | --- | --- |
| **KRAS mutated patients (zero-time shift)** | | | | **KRASwt patients (zero-time shift)** | | | |
| **Cluster** | **Centroid** | **Size, %** | **No. patients, %** | **Cluster** | **Centroid** | **Size, %** | **No. patients, %** |
| 3 | 0.88 | 52 | 57 | 3 | 0.89 | 61 | 66 |
| 2 | 0.08 | 19 | 22 | 2 | 0.22 | 18 | 21 |
| 1 | -0.83 | 29 | 31 | 1 | -0.90 | 21 | 22 |
| **Study 045** | | | | | | | |
| **KRAS mutated patients (zero-time shift)** | | | | **KRASwt patients (zero-time shift)** | | | |
| **Cluster** | **Centroid** | **Size, %** | **No. patients, %** | **Cluster** | **Centroid** | **Size, %** | **No. patients, %** |
| 3 | 0.92 | 25 | 29 | 3 | 0.99 | 63 | 71 |
| 2 | 0.05 | 25 | 29 | 2 | 0.63 |  |  |
| 1 | -0.76 | 50 | 57 | 1 | -0.43 | 38 | 43 |
| **OPUS** | | | | | | | |
| **KRAS mutated patients (zero-time shift)** | | | | **KRASwt patients (zero-time shift)** | | | |
| **Cluster** | **Centroid** | **Size, %** | **No. patients, %** | **Cluster** | **Centroid** | **Size, %** | **No. patients, %** |
| 3 | 0.93 | 61 | 66 | 3 | 0.89 | 85 | 85 |
| 2 | 0.14 | 14 | 14 | 2 | 0.18 | 6 | 8 |
| 1 | -0.96 | 25 | 24 | 1 | -0.66 | 9 | 12 |

No, number of; CC, cross-correlation coefficients. If cluster centroid value was above 0.35, CCs from that cluster were considered to show similar dynamics and included into cluster 3. Note that patient percentages accounted for more than 100%, because a single patient can have lesion pairs in different clusters. APEC study did not present any KRAS mutated patients, so it was not included in this comparison.

**Inter-class analysis results: SLD versus SOPD for each study**

Table SII. Inter-class analysis results based on SLD and SOPD metrics are reported side-by-side for each study where the tumor size was measured bidimensionally.

| **CRYSTAL** | | | | | | | |
| --- | --- | --- | --- | --- | --- | --- | --- |
| **SLD (zero-time shift)** | | | | **SOPD (zero-time shift)** | | | |
| **Cluster** | **Centroid** | **Size, %** | **No. patients, %** | **Cluster** | **Centroid** | **Size, %** | **No. patients, %** |
| 3 | 0.88 | 56 | 62 | 3 | 0.89 | 60 | 66 |
| 2 | 0.15 | 19 | 21 | 2 | 0.18 | 17 | 21 |
| 1 | -0.86 | 25 | 26 | 1 | -0.86 | 22 | 23 |
| **Study 45** | | | | | | | |
| **SLD (zero-time shift)** | | | | **SOPD (zero-time shift)** | | | |
| **Cluster** | **Centroid** | **Size, %** | **No. patients, %** | **Cluster** | **Centroid** | **Size, %** | **No. patients, %** |
| 3 | 0.87 | 44 | 50 | 3 | 0.87 | 50 | 50 |
| 2 | -0.04 | 19 | 21 | 2 | 0.20 | 19 | 21 |
| 1 | -0.68 | 38 | 43 | 1 | -0.67 | 31 | 36 |
| **OPUS** | | | | | | | |
| **SLD (zero-time shift)** | | | | **SOPD (zero-time shift)** | | | |
| **Cluster** | **Centroid** | **Size, %** | **No. patients, %** | **Cluster** | **Centroid** | **Size, %** | **No. patients, %** |
| 3 | 0.91 | 72 | 74 | 3 | 0.94 | 84 | 84 |
| 2 | 0.15 | 10 | 11 | 2 | 0.38 |  |  |
| 1 | -0.89 | 17 | 18 | 1 | -0.88 | 16 | 20 |

No, number of; CC, cross-correlation coefficients. SLD, sum of longest diameters. SOPD, sum of the products of diameters. If cluster centroid value was above 0.35, CCs from that cluster were considered to show similar dynamics and included into cluster 3. APEC study was not analyzed because it measured only the longest diameter value, so SOPD could not be obtained.

**Intra-class analysis results**

Table SIII. Intra-class analysis results for cetuximab arm patients are reported for each considered tumor tissue.

| **Class** | **No iTLs** | **No. patients** | **% CC in cluster 3 at zero shift** | **% CC in cluster 1 at maximum CC** | **Size reduction in cluster 1 with maximum CC vs zero shift, % CC** |
| --- | --- | --- | --- | --- | --- |
| Liver | 2244 | 602 | 79 | 4 | -4 |
| Lung | 340 | 111 | 88^a^ | 9 | -4 |
| Lymph node | 267 | 96 | 77^b^ | 14 | -9 |
| Other | 139 | 54 | 71^b^ | 14 | -15 |

No, number of; CC, cross-correlation coefficients. Cluster 3, cluster which contains CC values close to 1, showing similar lesion dynamics. Cluster 1, cluster which contains CC values close to -1, showing different lesion dynamics.

^a^ % CC corresponding to both clusters 2 and 3, as cluster 2 presented a large centroid (0.40) and high CC values.

^b^ % CC corresponding to the cluster 2, only two clusters were considered needed in this class based on the elbow method described in the Methods section.

Supplementary figures legends


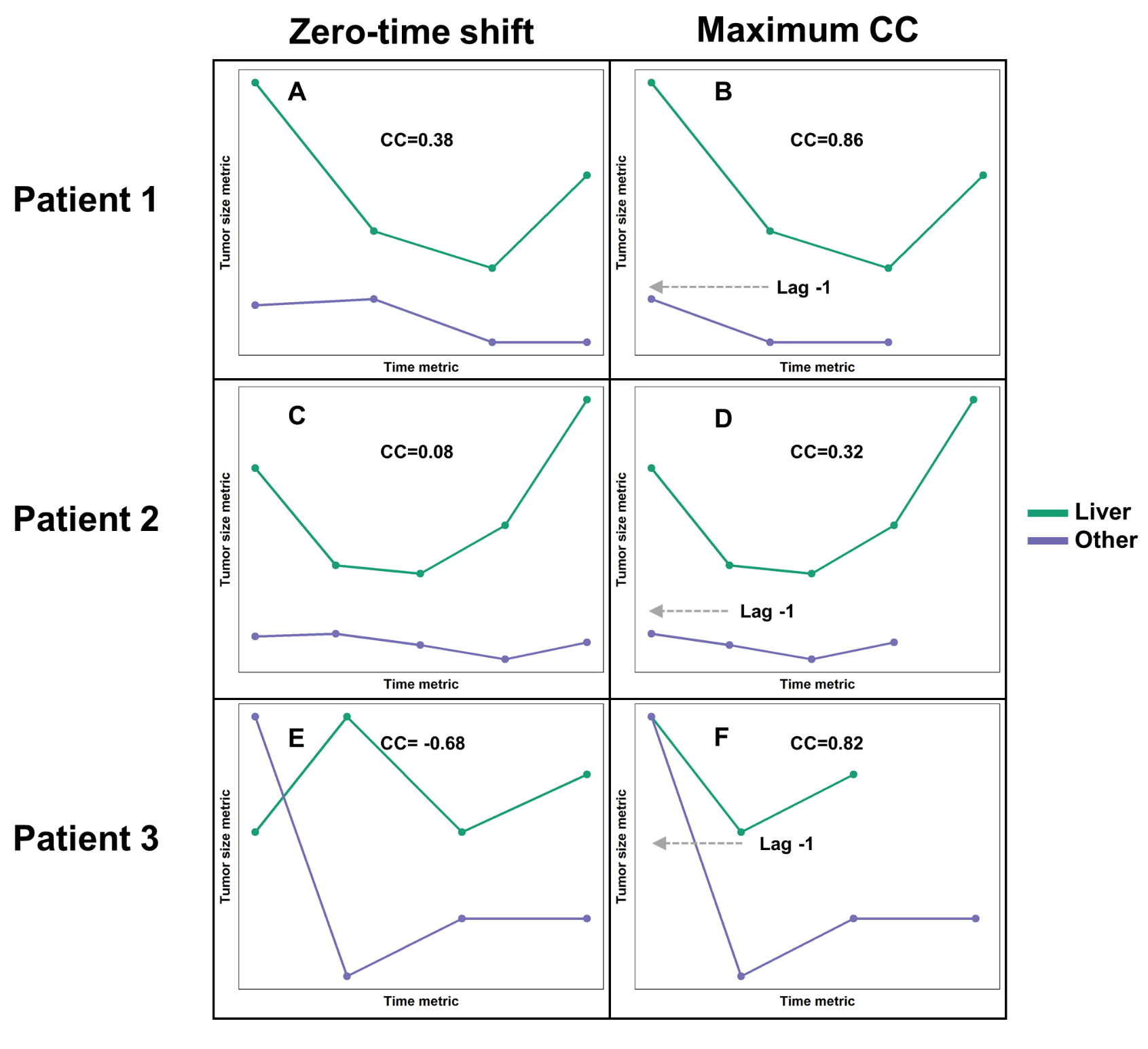


Figure S1. Several examples of patients’ lesion dynamics for different CCs value at zero-time shift. The reference lesion was the largest lesion at baseline; the other lesions were shifted to calculate the maximum cross-correlation coefficient (CC). Figures 1A, 1C and 1E show class-related target lesions (cTLs) at zero-time shift. The CCs show similar, undefined or opposite TS dynamics. When the maximum CCs were computed (figures 1B, 1D and 1F), similar lesion dynamics were suggested at -1 time shift.
