# Supplementary figures and images for: Machine Learning Analysis of Individual Tumor Lesions in Four Metastatic Colorectal Cancer Clinical Studies: Linking Tumor Heterogeneity to Overall Survival
Source: AAPS J. 2020 Mar 16;22(3):58. doi: 10.1208/s12248-020-0434-7 (PMC7078147; doi:10.1208/s12248-020-0434-7)

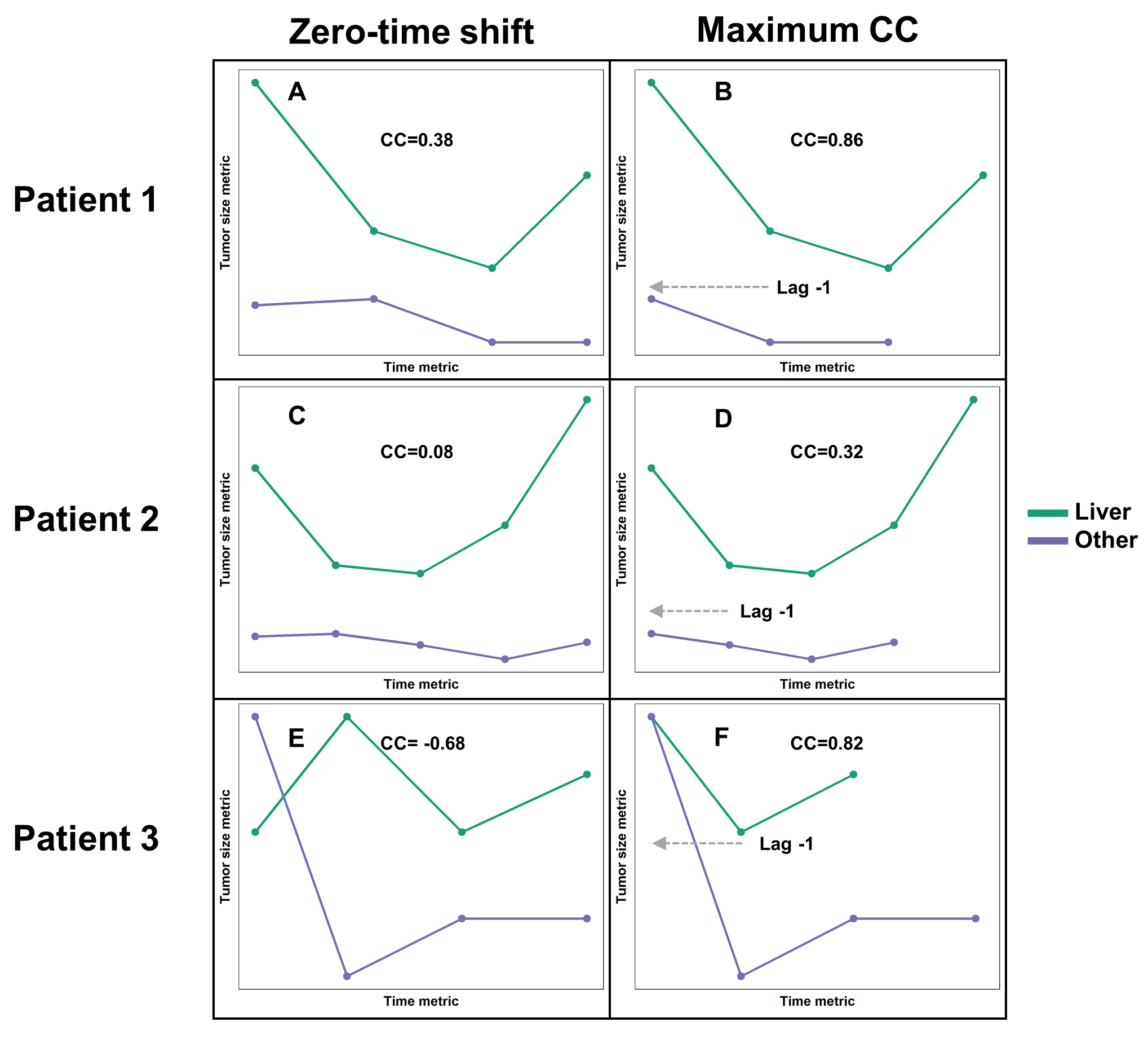

Supplement: Supplementary file 2 — (PNG 301 kb) [file 12248_2020_434_Fig5_ESM.png]

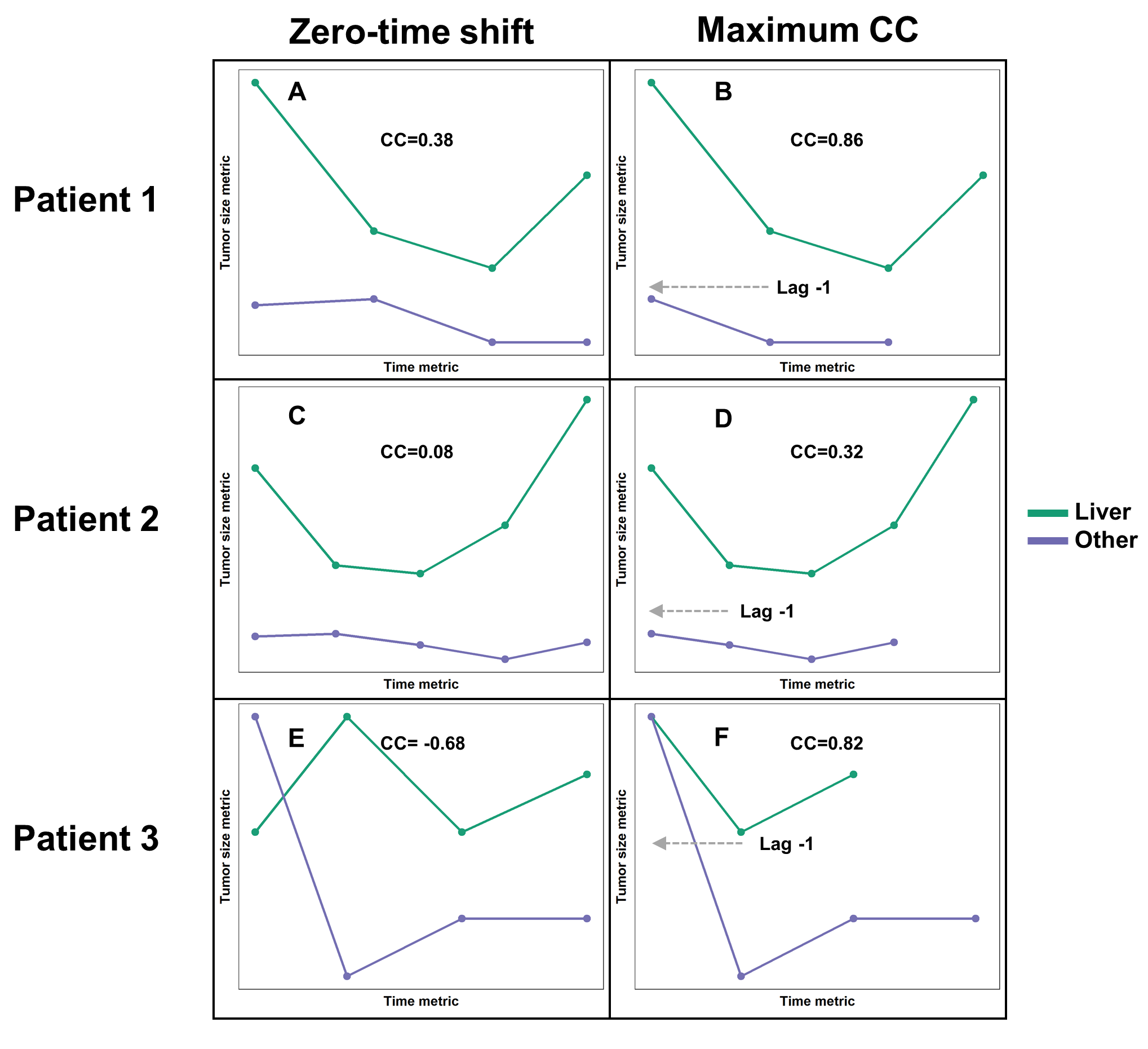

Supplement: Supplementary file 3 — High Resolution (TIF 657 kb) [file 12248_2020_434_MOESM2_ESM.tif]
